# Detection of Vaccine-Derived Spike Protein Associated with Immune Cell Infiltration in the Heart and Liver: A Report of Two Cases

**DOI:** 10.3390/cells15110978

**Published:** 2026-05-26

**Authors:** Michael Mörz, Alberto Donzelli, Robert Llewellyn Clancy, Shigetoshi Sano, Masanori Fukushima, Panagis Polykretis

**Affiliations:** 1 Institute of Pathology “Georg Schmorl”, Municipal Hospital Dresden-Friedrichstadt Site, 01067 Dresden, Germany; michael.moerz@klinikum-dresden.de; 2 Independent Medical Scientific Commission (CMSi), 20122 Milano, Italy; 3 “Allineare Sanità e Salute” Foundation, 20131 Milano, Italy; 4 School of Medicine and Public Health, University of Newcastle, Newcastle 2000, Australia; 5 Department of Dermatology, Kochi Medical School, Kochi University, Nankoku-shi 783-8505, Japan; 6 Sano Dermatology Clinic, Nishinomiya 663-8184, Japan; 7 Learning Health Society Institute, Nagoya 450-0003, Japan

**Keywords:** COVID-19 genetic vaccines, autoimmunity, adverse reactions, inflammation, immune cell recruitment

## Abstract

**Highlights:**

**What are the main findings?**

- Immunohistochemical analysis of two autopsy cases detected the vaccine-derived SARS-CoV-2 spike protein within areas of histio-lymphocytic myocarditis and hepatic inflammation.
- The consistent absence of the viral nucleocapsid protein confirmed that the spike protein expression was vaccine-induced, effectively ruling out a natural viral infection in the affected organs.

**What are the implications of the main findings?**

- The findings demonstrate that COVID-19 vaccine components can distribute systemically, leading to the off-target expression of the spike protein in tissues far beyond the injection site.
- The localized synthesis of vaccine-derived antigens by host cells can trigger targeted immune-cell recruitment and tissue inflammation, providing crucial mechanistic insights into post-vaccination adverse events.

**Abstract:**

The rapid development and deployment of COVID-19 genetic vaccines have raised significant concerns regarding their safety and potential to trigger immune reactions against self-tissues. This paper provides a comprehensive histopathologically supported analysis of how the synthesis of the vaccine-derived spike protein can trigger such reactions beyond the injection site, characterized by robust immune cell recruitment. We examine these immune responses based on histopathological evidence that delineates a pattern consistent with self-directed immune activity, including vaccine-associated myocarditis. In this regard, we report two representative cases, marked by immune-cell infiltration, triggered by the synthesis of the vaccine-derived spike protein in the myocardium and in the liver, respectively. Additionally, we provide a detailed characterization of the process and the immune cells involved in these reactions, based on histopathological findings. Understanding these mechanisms is essential for accurately assessing the potential implications of these vaccination technologies on human health. By emphasizing the need for further research into the pharmacokinetics and off-target effects of COVID-19 genetic vaccines, this paper aims to deepen our understanding of their safety profiles and inform future vaccine development.

## 1. Introduction

The global response to the COVID-19 pandemic has led to the rapid development and deployment of genetic vaccines designed to instruct human cells in producing the viral protein known as the SARS-CoV-2 spike protein [1]. While these vaccines have been praised for their safety and effectiveness, emerging research indicates that they may cause severe inflammatory responses against self-tissues [2,3]. Such responses occur when the immune system identifies the vaccine-derived protein as a foreign entity, subsequently targeting the host cells that synthesize it [3]. This mechanism was first proposed by Dr. Polykretis, who emphasized the necessity of biodistribution studies to evaluate the risks associated with the spread of vaccine-derived genetic material beyond the injection site [3,4,5,6].

This paper aims to describe the histopathological profile resulting from the recruitment of immune cells targeting host cells that produce the vaccine-derived spike protein, along with the associated inflammatory response.

In order to fully understand the mechanistic details underlying such vaccine-induced reaction, we have to introduce the antigen presentation process. In human cells, this process is carried out by two protein complexes, the Major Histocompatibility Complex I and II (MHC I and II), which are essential for cell-mediated immunity [7,8]. MHC I is located on the membrane of all nucleated cells and presents to CD8^+^ cytotoxic T-lymphocytes fragments of endogenous antigens, generated upon the proteasomal degradation of intracellular proteins. This mechanism allows the immune system to continuously monitor the proteosynthetic activity of all nucleated cells to determine whether a cell is producing mutant, viral, or non-self proteins. The MHC II displays fragments of exogenous antigens that have been phagocytized throughout the body to CD4^+^ T-helper lymphocytes, and it is found on the membranes of professional antigen-presenting cells (APCs), such as dendritic cells and macrophages. APCs intrinsically undergo programmed cell death as part of their role in presenting antigens to immune cells, necessitating a continuous turnover to maintain effective immune responses [9]. When the immune system recognizes a foreign antigen presented either by MHC I on a somatic cell or by MHC II on an APC, it targets the respective cell, triggering a response that leads to its death. While APCs undergo continuous turnover as part of their physiological role, the destruction of somatic cells can result in significant symptomatic damage, depending on the affected tissue. This mechanism raises serious concerns, since it has been demonstrated that the vaccine-derived genetic material can enter systemic circulation and reach not only tissues distant from the injection site but also bodily secretions such as breast milk [10].

Despite the potential risk of triggering reactions against self-tissues synthesizing the vaccine-derived spike protein, inherent in the immunization mechanism of genetic vaccines [3,4], these pharmaceutical products have been widely implemented and administered indiscriminately, including to pregnant women and age groups with an extremely low probability of dying from COVID-19 [11].

## 2. Materials and Methods

### 2.1. Patient Data and Clinical History

This study describes the histopathological findings from two patients with distinct clinical backgrounds and vaccination histories:

- **Myocarditis case**: A 72-year-old male who died from cryptogenic organizing pneumonia (COP), with histio-lymphocytic myocarditis identified as the main pathological condition. His vaccination history included two doses of AstraZeneca (April 2021, lot number: ABW2586; July 2021, lot number: 210094), one dose of Moderna (December 2021, lot number: 042G12A), and a booster dose of Pfizer/BioNTech (November 2022, 15 μg Original/Omicron BA.4-5). No known COVID-19 infection was recorded in the medical history.
- **Hepatitis case**: An 86-year-old patient, with no known liver disease, who died from decompensated heart failure. The major concomitant condition was chronic obstructive pulmonary disease (COPD). The vaccination history included three doses of the Pfizer/BioNTech vaccine administered in March 2021 (lot number: ER2659 and EZT3674, for the first and second dose, respectively) and November 2021 (lot number: 1F1023A). No documented COVID-19 infection was reported in the medical history.

Due to the retrospective post-mortem nature of these histopathological evaluations, comprehensive ante-mortem clinical laboratory data (such as specific serum biomarkers or functional assessments) were unavailable for review. Consequently, the scope of this clinical history is limited to the available records, and the present study focuses strictly on the post-mortem histopathological and immunohistochemical tissue evaluations.

### 2.2. Tissue Collection and Processing

Post-mortem cardiac tissue and liver samples were collected and fixed in 10% neutral-buffered formalin, routinely processed, and embedded in paraffin. Sections of 5 μm thickness were prepared and stained with hematoxylin and eosin (H&E) for routine histopathological examination.

### 2.3. Immunohistochemical Analysis

Immunohistochemical detection of the SARS-CoV-2 spike protein was conducted following a protocol adapted from Mörz [12]. Formalin-fixed paraffin-embedded tissue sections were processed on a fully automated immunostaining platform (Ventana BenchMark, Roche, Basel, Switzerland). Antigen retrieval was carried out with Ultra CC1 solution (Roche Ventana) to unmask epitopes. Primary antibodies utilized included those targeting SARS-CoV-2 spike subunit 1 (ProSci, San Diego, CA, USA; clone 9083) and nucleocapsid protein (ProSci, clone 35-720), both applied at a dilution of 1:500 and incubated for 32 min. Additionally, immune cell markers were applied to characterize T-lymphocyte and monocytic populations as follows: CD3 (Zytomed Systems, Berlin, Germany; clone ZM-45, dilution 1:200) was incubated for 30 min; CD4^+^ (Zytomed Systems, Berlin, Germany; clone SP35, RTU) was incubated for 32 min; CD8^+^ (Zytomed, clone C8/144B, RTU) was incubated for 20 min; and CD68 (Dako, Carpinteria, CA, USA; clone PG-M1, dilution 1:100) was incubated for 20 min. Following incubation with primary antibodies, detection was performed using standardized visualization systems. Slides were examined via light microscopy (Nikon Corporation, Tokyo, Japan), and representative images were acquired using a Moticam 3 Motic^®^ (Motic Europe, Barcelona, Spain) camera system. Immunohistochemical staining for SARS-CoV-2 nucleocapsid was consistently negative across all samples, indicating an absence of detectable viral nucleocapsid antigen in the tissues examined (see Supplementary Materials).

### 2.4. Control Sample Preparation

The immunohistochemical differentiation between vaccine-induced spike protein expression and natural SARS-CoV-2 infection (using spike and nucleocapsid antibodies) was performed according to the protocol previously described by Mörz [12]. Briefly, ovarian cancer cell lines OVCAR-3 and SK-OV3 (CSL Cell Lines Service, Heidelberg, Germany) were cultured in flat-bottom 75 cm^2^ flasks (Cellstar, Greiner Bio-One, Kremsmünster, Austria) in DMEM/HAMS-F12 medium supplemented with Glutamax (Sigma-Aldrich, St. Louis, MO, USA), 10% fetal calf serum (Gibco, Thermo Fisher Scientific, Shanghai, China), and gentamicin (20 μg/mL; Gibco) at 37 °C in a humidified incubator with 5% CO_2_ until reaching approximately 70% confluence. For transfection, the medium was completely removed, and cells were incubated for one hour in 2 mL fresh medium containing vaccine solutions directly from the original bottles, diluted 1:500 for BNT162b2 (Pfizer/BioNTech) and 1:100 for mRNA-1273 (Moderna) and Vaxzevria (AstraZeneca) vaccines. Following this incubation, 15 mL fresh medium was added, and cells were cultured for an additional three days until confluence. Afterward, the culture medium was discarded, cells were washed twice with phosphate-buffered saline (PBS), detached using 1 mL 0.25% Trypsin-EDTA (Gibco, Thermo Fisher Scientific, Shanghai, China), neutralized with 10 mL PBS containing 10% FCS, and centrifuged at 280× *g* for 10 min; this wash step was repeated twice. Cell pellets were fixed overnight at 8 °C in 2 mL 4% formalin in PBS, washed once with PBS, then resuspended in 200 μL PBS and combined with 400 μL of 2% agarose in PBS preheated to approximately 40 °C. The resulting suspension was immediately transferred into small 1 cm molds for solidification. The agarose-embedded cell pellets were then stored in 4% formalin/PBS and processed for routine paraffin embedding alongside tissue samples, serving as internal positive and negative controls for the immunohistochemical procedures.

## 3. Results

### 3.1. Myocarditis Case

**Figure 1:**
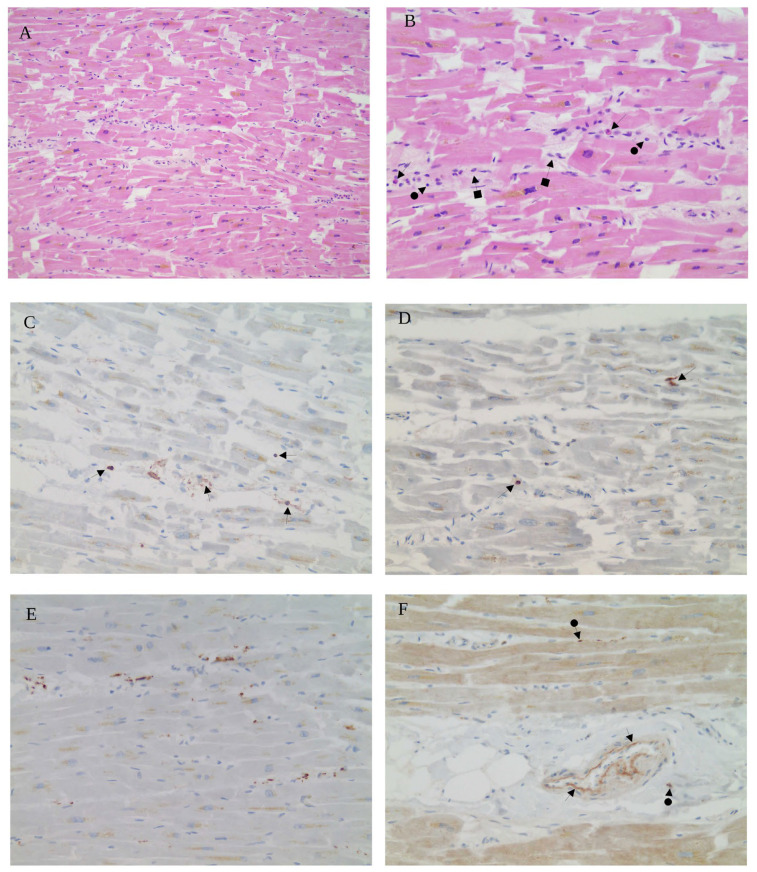
(**A**) H&E staining at 100× showing borderline histio-lymphocytic myocarditis with single-cell necrosis, capillary proliferation, and contraction band necrosis. (**B**) H&E (200×) demonstrating myocardial infiltration by eosinophils (arrows), macrophages (arrows with square), and lymphocytes (arrows with dot). (**C**) CD4 immunostaining (DAB, 200×) revealing abundant interstitial CD4^+^ T-cells (arrows). (**D**) CD8 immunostaining (DAB, 200×) showing fewer CD8^+^ cytotoxic T-lymphocytes (arrows). (**E**) CD68 staining (DAB, 200×) highlighting predominant CD68^+^ macrophages in the interstitium. (**F**) SARS-CoV-2 spike subunit 1 immunostaining (DAB, 200×) with positive staining of endothelial cells (arrows) and infiltration of inflammatory cells with a high content of CD68 macrophages (arrows with dot), including visible inflammatory cell penetration of the vessel wall.

Histopathological examination of myocardial tissue revealed features consistent with borderline histio-lymphocytic myocarditis. H&E staining demonstrated focal areas of single-cell necrosis, notable capillary proliferation, and contraction band necrosis (Figure 1A). The inflammatory infiltrate was heterogeneous, predominantly composed of eosinophils, macrophages, and lymphocytes dispersed throughout the myocardial interstitium (Figure 1B). The immunohistochemical analysis showed a substantial population of CD4^+^ T lymphocytes distributed throughout the interstitium (Figure 1C), while CD8^+^ cytotoxic T-lymphocytes were also present but in relatively lower numbers (Figure 1D). The majority of infiltrating immune cells were CD68^+^ macrophages, underscoring their prominent role in the inflammatory milieu (Figure 1E). Importantly, immunostaining for the SARS-CoV-2 spike subunit 1 protein revealed positive expression in endothelial cells lining the myocardial vasculature as well as in infiltrating inflammatory cells (Figure 1F). Notably, the presence of vaccine-derived spike protein in the affected cardiac tissue appears to trigger activation of the endothelium, promoting the infiltration of inflammatory cells through the vascular wall and thereby contributing to the local immune response. Immunohistochemistry for SARS-CoV-2 nucleocapsid protein was negative, confirming that the myocardial inflammation was not associated with the presence of the virus (Supplementary Materials, Figure S1).

### 3.2. Hepatitis Case

**Figure 2:**
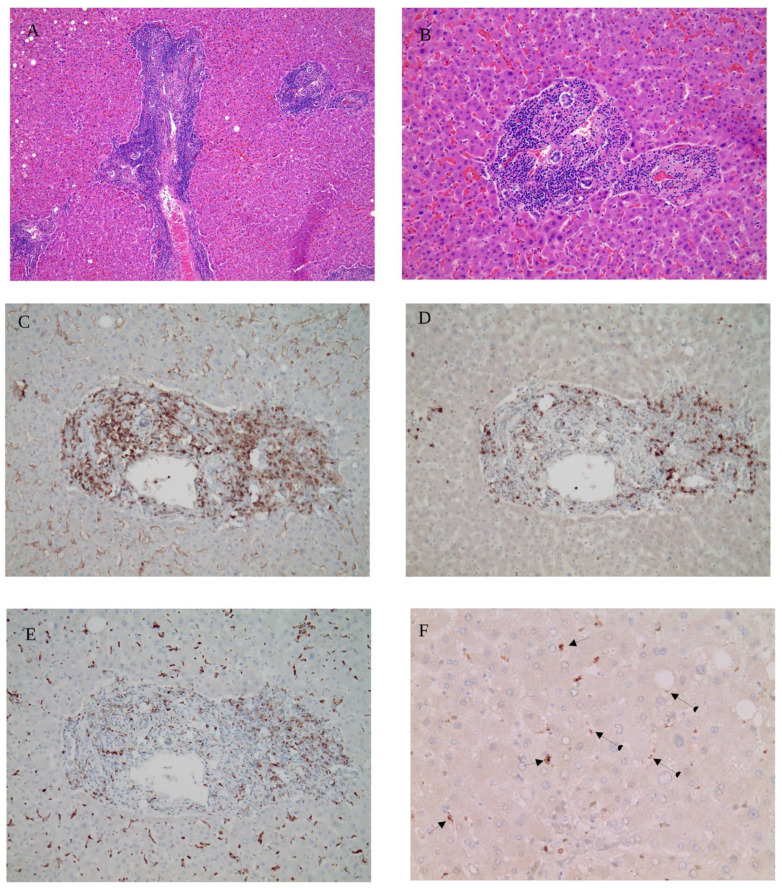
(**A**) H&E staining at 100× showing enlarged portal tracts infiltrated by lymphocytes, plasma cells, and eosinophilic granulocytes, with interface activity and bile duct proliferation; sinusoidal lymphocyte infiltration is also visible. (**B**) Higher magnification H&E (200×) of portal tract inflammation. (**C**) CD4 immunostaining (DAB, 200×) demonstrating numerous CD4^+^ T-helper lymphocytes in portal areas. (**D**) CD8 staining (DAB, 200×) showing fewer CD8^+^ cytotoxic T-lymphocytes compared to CD4^+^ cells in the portal tract. (**E**) CD68 immunostaining (DAB, 200×) highlighting prominent macrophages/Kupffer cells. (**F**) SARS-CoV-2 spike subunit 1 staining (DAB, 400×) demonstrating vaccine-derived spike protein presence in cells morphologically and anatomically consistent with Kupffer cells (arrows) and sinusoidal endothelial cells (arrow with dot).

The histological analysis of liver tissue demonstrated features consistent with chronic hepatitis in the context of primary biliary cirrhosis. H&E staining revealed markedly enlarged portal tracts extensively infiltrated by dense accumulations of lymphocytes, plasma cells, and eosinophilic granulocytes, accompanied by evident interface activity and bile duct proliferation (Figure 2A). Higher magnification further emphasized the pronounced inflammatory infiltrate within the portal tract and extending into the adjacent hepatic parenchyma (Figure 2B). The immunohistochemical characterization showed a prominent presence of CD4^+^ T-helper lymphocytes populating the portal areas (Figure 2C), whereas CD8^+^ cytotoxic T-lymphocytes were comparatively fewer, indicating a predominance of CD4^+^ over CD8^+^ T-cell subsets (Figure 2D). CD68 immunostaining highlighted a significant infiltration of inflammatory cells with a high content of CD68^+^ macrophages within the portal regions, suggesting the activation of Kupffer cells and monocyte-derived macrophages (Figure 2E). Importantly, SARS-CoV-2 spike subunit 1 protein was detected in CD68^+^ cells and sinusoidal endothelial cells (Figure 2F), implicating these cell types as potential reservoirs of vaccine-derived spike protein expression, although double staining to confirm co-localization was not performed. This localized vaccine-derived spike protein presence correlates with the observed inflammatory infiltrate and interface hepatitis characteristic of primary biliary cirrhosis. Immunohistochemistry for SARS-CoV-2 nucleocapsid protein showed no staining, indicating the absence of viral infection in the hepatic tissue (Supplementary Materials, Figure S2).

## 4. Discussion

Immune reactions targeting self-tissues have been observed in the context of SARS-CoV-2 infection and following genetic vaccination. SARS-CoV-2 infection can induce autoimmune phenomena, as documented by Sacchi et al. [13], Bonometti et al. [14], Zhou et al. [15], and Vlachoyiannopoulos et al. [16]. These studies were conducted prior to the widespread use of genetic vaccines, ensuring that the effects of infection and vaccination did not overlap in the patient cohorts analyzed. In severely ill patients, innate immune hyperactivation leads to a cytokine storm characterized by elevated levels of inflammatory mediators such as interleukin-6. This hyperinflammatory state disrupts microcirculation and can cause shock and acute respiratory distress syndrome. Importantly, cytokines released during the storm may drive autoinflammatory reactions and trigger autoimmunity, potentially through the activation of pre-existing autoreactive B cell clones or molecular mimicry mechanisms [16].

In contrast, immune reactions triggered by vaccination are driven by a different mechanism. The genetic vaccines against COVID-19 contain instructions that enable human cells to use their own machinery to synthesize the SARS-CoV-2 spike protein, the viral antigen that triggers an immune response [1]. The spike protein is not released from the cells, as claimed in some studies [17]; instead, it is presented on the surface of cells that have translated the vaccine mRNA, as reported in the Comirnaty assessment report by the European Medicines Agency (EMA) [18]. Therefore, antigen presentation likely occurs in two forms: either as the full protein expressed on the cell surface, enabling recognition by B cells and CD4^+^ T-helper cells, or as peptide fragments bound to MHC class I molecules, which present internal antigens to CD8^+^ T lymphocytes [4]. Thus, even if the spike protein were to be released, the autoimmune mechanism could still be activated due to the fragments presented by the MHC I, as noted by Polykretis [4]. In both scenarios, all cells presenting vaccine-derived antigens are perceived as threats by the immune system and are targeted for destruction [3,4,5,6]. Regarding the immune response against self-tissues synthesizing the vaccine-derived spike protein, this reaction fits within the broader definition of autoimmunity as classically described: “*a condition in which the body produces an immune response against its own tissue constituents*” [19]. This broader perspective highlights the involvement of self-cells and -tissues targeted by the immune system. However, from a molecular viewpoint, the vaccine-derived spike protein is not an autoantigen, as it is a foreign viral protein produced by host cellular machinery rather than a native self-protein. This distinction aligns with narrower molecular definitions of autoimmunity that focus exclusively on immune reactivity toward endogenous self-antigens. Importantly, regardless of definitional nuances, tissues that would be normally healthy and vital may become unintended targets of immune-mediated damage following vaccination due to intracellular expression of the spike protein. This risk is heightened by the broad biodistribution of LNPs, which can deliver genetic material to tissues not typically reached by the virus during natural infection.

A recent review summarized the histopathological findings from biopsies and autopsies of patients who developed severe inflammatory reactions against self-tissues following vaccination [3], thereby providing validation for the mechanism previously predicted by Dr. Polykretis [4]. Since the publication of the aforementioned review, several new studies have emerged, further expanding our understanding of this mechanism. In the autopsy study by Krauson et al., the vaccine mRNA was detected by RT-qPCR in axillary lymph nodes and in the heart of patients who died within 30 days of vaccination [20]. Koizumi et al. describe three cases of unexplained cardiac arrest with multiple micro-scars in key myocardial regions linked to arrhythmias, all in patients with COVID-19 booster vaccinations [21]. The scars likely result from immune-mediated microvascular injury related to spike protein expression, creating a substrate for fatal arrhythmias. A retrospective study of 19 hemorrhagic stroke cases, examined the long-term presence of SARS-CoV-2 spike protein in brain tissues after mRNA vaccination [22]. The spike protein was detected in the cerebral arteries of 43.8% of vaccinated patients, sometimes up to 17 months post-vaccination. In situ hybridization confirmed both vaccine- and virus-derived spike mRNA in some cases, but no evidence of ongoing viral infection was found. The findings suggest that the mRNA vaccine-derived spike protein can persist in cerebral vessels for extended periods. Similarly, Geeraerts et al. examined fatal cases of vaccine-induced immune thrombotic thrombocytopenia (VITT) following adenoviral-vectored COVID-19 vaccination [23]. Immunohistochemical analysis revealed the vaccine-derived spike protein within nucleated cells (predominantly neutrophils) of cerebral venous thrombi, as well as in adjacent vessel walls. The authors attribute this distant localization either to the local translation of the systemically distributed vaccine vector or to the cellular uptake of circulating immune zcomplexes. These findings further corroborate that vaccine-derived components can reach and trigger immune-mediated reactions in distant anatomical regions. Additional reports reinforce the concern over prolonged vaccine antigen presence. An alarming study by Sano et al. describes the case of a 53-year-old woman who developed vesiculopapular lesions on her arm and papulonecrotic lesions on her legs following her third dose of the Pfizer/BioNTech vaccine [24]. Immunohistochemical analysis of the lesions revealed the presence of the vaccine-derived spike protein within eccrine sweat gland/duct, through which sweat is excreted, and the basal layers of the epidermis—persisting up to 15 months after vaccination. The authors conclude that such a phenomenon could be explained by the prolonged persistence of vaccine-derived genetic material, or even more concerning, by its possible integration into the genome of the patient’s skin cells. All the aforementioned histopathological studies share a common pattern marked by the off-target synthesis of the vaccine-derived spike protein, which leads to the recruitment of immune cells in the affected tissue.

**Figure 3:**
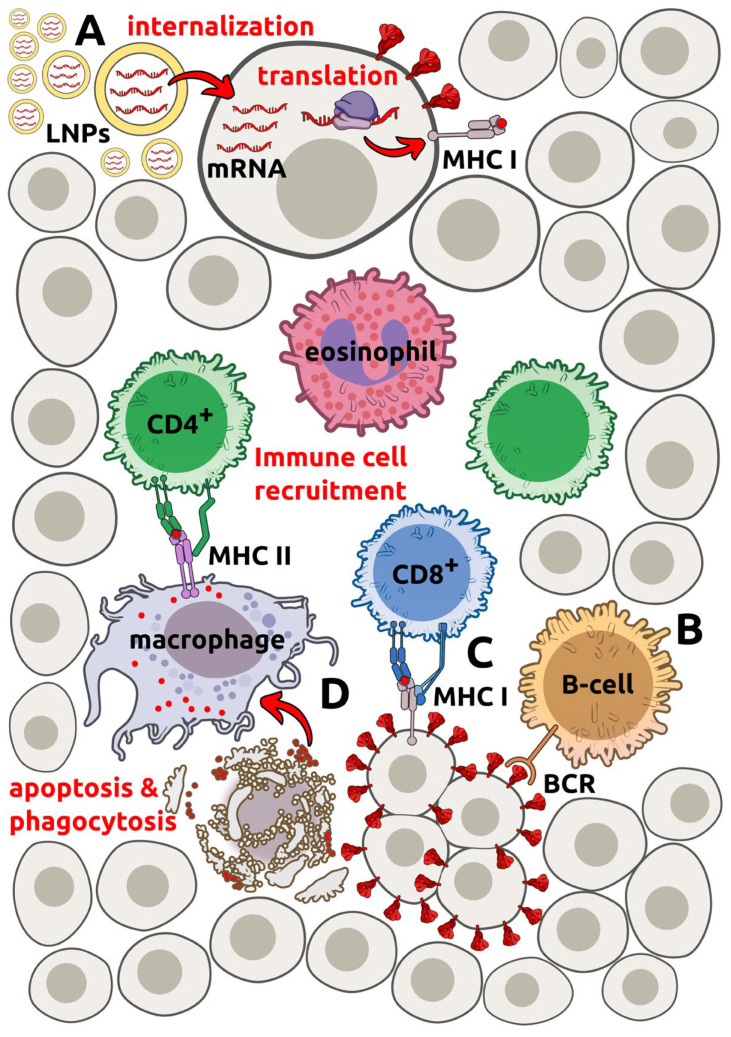
Schematic representation of the vaccine-derived autoimmune inflammatory reaction. (**A**) The LNPs deliver the vaccine mRNA into the endothelial cells, where it is translated by ribosomes, and the resulting vaccine-derived spike protein is then presented both on the cell membrane in its full form and as peptide fragments bound to MHC I. (**B**) An endothelial cell presenting the vaccine-derived spike protein to the B-cell receptor (BCR) of a B cell. (**C**) The MHC I of an endothelial cell presents peptides, derived from proteasomal degradation of endogenously synthesized spike protein, to the T-cell receptor (TCR) of a CD8^+^ lymphocyte. (**D**) The MHC class II molecule of a macrophage presents peptides derived from the spike protein, following apoptosis and subsequent phagocytosis of an endothelial cell to the T-cell receptor (TCR) of a CD4^+^ lymphocyte.

The present study provides further histopathological evidence of immune-mediated injury affecting both myocardial and hepatic tissues following genetic vaccination. In the myocarditis case, we observed a mixed inflammatory infiltrate mediated by CD4^+^ T-helper lymphocytes, CD8^+^ lymphocytes and CD68^+^ macrophages within the myocardial interstitium, accompanied by signs of endothelial involvement and the presence of the SARS-CoV-2 spike subunit 1 protein within vascular endothelial cells and infiltrating macrophages (Figure 1). Similarly, in the hepatitis case, portal and periportal regions were densely infiltrated by lymphocytes, plasma cells, eosinophils, and a preponderance of CD4^+^ T-helper cells, with notable macrophage/Kupffer cell activation and prominent spike protein localization within these cells as well as sinusoidal endothelial cells (Figure 2). Our analysis incorporated direct immunohistochemical detection of the SARS-CoV-2 spike protein, allowing us to identify specific cellular reservoirs of vaccine-derived antigen within affected tissues. Notably, the detection of spike protein in both endothelial and immune cells in situ further supports the concept of off-target antigen expression as a driver of localized immune activation and tissue injury. Taken together, the immune cell profiles and the vaccine-derived spike protein distribution observed in our cases reveal a consistent pattern of CD4^+^ T-cell and macrophage predominance at sites of tissue injury, reflecting mechanisms involved in both autoimmune myocarditis and chronic immune-mediated hepatitis. Importantly, our results establish a link between the presence of vaccine-derived spike protein in tissues distant from the injection site and the observed inflammatory reactions, supporting earlier mechanistic predictions and adding to the growing evidence of a pathogenic pathway for off-target tissue injury following genetic vaccination. Our findings are consistent with the existing evidence [25,26,27], which linked post-vaccination deaths to immune-mediated myocardial inflammation, primarily driven by CD4^+^ T-cell infiltration, in the absence of active infection or prior heart disease. This mechanism is schematically represented in Figure 3.

Importantly, the risk of developing myocarditis and pericarditis following mRNA COVID-19 vaccination has been acknowledged by the CDC [28]. In this regard, it is often claimed that autoimmune inflammatory reactions, such as myocarditis and pericarditis, are extremely rare and are more likely to be triggered by infection than by vaccination [29]. However, a large retrospective cohort study performed by Tuvali and co-authors, found that there is no statistically significant increase in the incidence of myocarditis or pericarditis in unvaccinated subjects after SARS-CoV-2 infection [30]. Moreover, a very large observational study conducted in the OpenSAFELY-TPP database, including adolescents aged 12–15 years and children aged 5–11 years and comparing individuals receiving first vaccination with unvaccinated controls documented myocarditis (three cases) and pericarditis (nine cases) only in the vaccinated groups, with rates of 27 and 10 cases/million after first and second doses, respectively [31,32].

Furthermore, the prospective cohort study by Mansanguan and co-authors, done on 301 teenagers between the ages of 13 and 18, who had received two doses of the Pfizer/BioNTech vaccine, found that 29.24% of participants experienced cardiovascular complications, and 2.33% suffered myopericarditis [33]. Similarly, the prospective active surveillance study by Buergin and co-authors, conducted among 777 hospital employees scheduled to undergo booster vaccination with the Moderna vaccine, found that one in 35 recipients (2.8%) developed a vaccine-associated myocardial injury [34]. In the first aforementioned prospective surveillance study, myocardial injury markers were monitored both before and immediately after vaccination. Instead, in the second, the 2.8% rate may be even higher, because 40 participants (5.1%) had elevated high-sensitivity cardiac troponin T (hs-cTnT) concentration on day 3, but vaccine-associated myocardial injury was finally adjudicated only in 22 participants, because blood samples were not taken before vaccination, and for 18 subjects, an abnormal hs-cTnT was considered possibly associated with preexisting conditions. However, among a comparative group of presumably healthy individuals, only 1% of persons are expected to have increased hs-cTnT levels, using the sex-specific 99th percentile as the upper limit of normal [34]. The methodological rigor of active surveillance not only ensures high quality and reliability but also makes these findings highly representative of the real-world incidence of vaccine-associated cardiac events. They reconfirm that passive surveillance systems dramatically underestimate the reported adverse reactions after immunization. Indeed, active surveillance shows incidences of myopericarditis about one thousand times higher compared with several passive reporting systems (such as Italian Medicines Agency reports) [35].

## 5. Conclusions

Summarizing, it can be concluded that both severe natural SARS-CoV-2 infection and vaccination may provoke immune-mediated attacks against self-tissues, but these reactions exhibit distinct patterns that can be discriminated through immunohistochemistry. Severe natural infection may lead to cytokine storms due to innate immune hyperactivity, potentially triggering autoinflammatory reactions through pre-existing B cell clones or molecular mimicry [16]. Regarding COVID-19 genetic vaccines, such responses occur when the immune system identifies the vaccine-derived protein as a foreign entity, leading to robust immune cell recruitment against host cells that synthesize it. As demonstrated by several histopathological studies, this process occurs in several vaccine-induced conditions, such as myocarditis [3]. Critically, the off-target synthesis of non-self proteins acting as antigens is a fundamental issue that must be addressed for all genetic vaccines, both current and future. This harmful phenomenon may not be as rare as previously thought, as prospective active surveillance studies have shown a concerning incidence of myocardial injury, with one in 35 of those vaccinated experiencing such effects, as reported by Buergin et al. [34]. Given these findings, comprehensive pharmacokinetic and pharmacodynamic studies, along with rational harm–benefit assessments by age group, are essential to prevent further loss of life. Damage deriving from an anthropogenic action (like vaccination) has a far higher ethical severity with respect to a natural cause like a viral infection (assuming that SARS-CoV-2 is natural [36], which is a debated topic that does not pertain to this study). The implications of these immune responses are critical for understanding both the safety and long-term effects of COVID-19 vaccines. By examining these mechanisms and reviewing recent histopathological evidence, this paper aims to clarify the immune response mechanisms and their health implications, which is crucial for developing strategies to mitigate potential adverse effects and to guide future research and public health policies.

## Data Availability

The original contributions presented in this study are included in the article/Supplementary Materials. Further inquiries can be directed to the corresponding author(s).

## Supplementary Materials

The following supporting information can be downloaded at https://www.mdpi.com/article/10.3390/cells15110978/s1, Figure S1: Heart: Borderline histio-lymphocytic myocarditis, 100× magnification; Figure S2: Liver: Chronic hepatitis, 100× magnification.
