# Supplementary material for: Detection of Vaccine-Derived Spike Protein Associated with Immune Cell Infiltration in the Heart and Liver: A Report of Two Cases
Source: Cells. 2026 May 26;15(11):978. doi: 10.3390/cells15110978 (PMC13256085; doi:10.3390/cells15110978)
Supplement: Supplementary file 1 [file cells-15-00978-s001.zip › cells-4241764-supplementary.pdf]

Supplementary Material

Detection of Vaccine-Derived Spike Protein Associated with Immune Cell Infiltration in the Heart and Liver: A Report of Two Cases

Michael Mörz [1], Alberto Donzelli [2,3], Robert Llewellyn Clancy[4], Shigetoshi Sano [5,6], Masanori Fukushima [7] and Panagis Polykretis [2,3,*,†]

[1] Institute of Pathology "Georg Schmorl", Municipal Hospital Dresden-Friedrichstadt Site, 01067 Dresden, Germany; michael.moerz@klinikum-dresden.de
[2] Independent Medical Scientific Commission (CMSi), 20122 Milano, Italy; donzelli@sanitaesalute.it
[3] "Allineare Sanità e Salute" Foundation, 20131 Milano, Italy
[4] School of Medicine and Public Health, University of Newcastle, Newcastle 2000, Australia; robert.clancy181@gmail.com
[5] Department of Dermatology, Kochi Medical School, Kochi University, Nankoku-shi 783-8505, Japan; sano.derma@kochi-u.ac.jp
[6] Sano Dermatology Clinic, Nishinomiya 663-8184, Japan
[7] Learning Health Society Institute, Nagoya 450-0003, Japan; mfukushima@imrd.jp
* Correspondence: panagis.polykretis@gmail.com; Tel.: +39-02-857-825-65
† Current address: Ancestralize Ltd., H-2096 Üröm, Hungary.

Immunohistochemical exclusion of natural SARS-CoV-2 infection in inflamed tissues

To rule out a natural SARS-CoV-2 infection as the underlying cause of the observed organ inflammation, immunohistochemical staining specifically targeting the SARS-CoV-2 nucleocapsid protein was performed on the patients' tissues. Formalin-fixed, paraffin-embedded tissue sections from the heart (exhibiting borderline histio-lymphocytic myocarditis) and the liver (exhibiting chronic hepatitis) were processed using a fully automated immunostaining system (Ventana Benchmark, Roche). Tissues were incubated with the primary antibody against the nucleocapsid protein, and the reaction was evaluated using a standard DAB detection kit. Slides were examined under a light microscope (**Figures S1 and S2**).

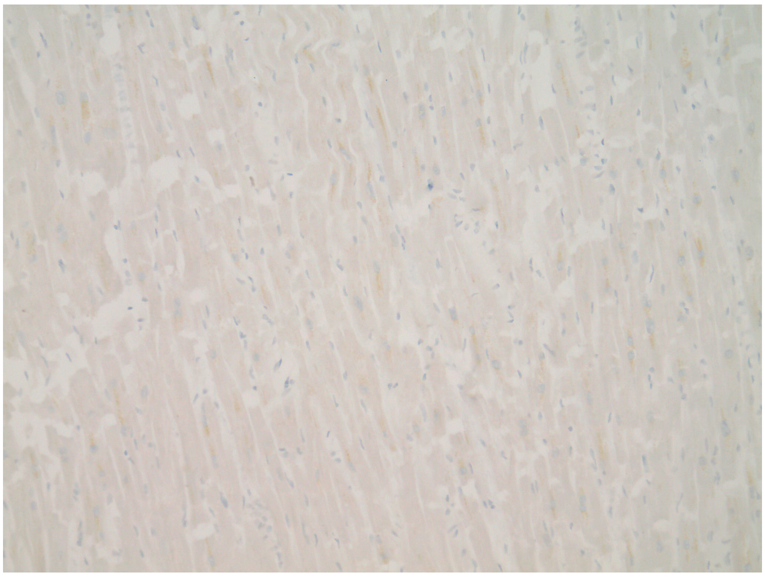

Figure S1. *Heart*: Borderline histio-lymphocytic myocarditis, 100x magnification. Immunohistochemistry for SARS-CoV-2 nucleocapsid protein shows no staining, confirming that the myocardial inflammation was not associated with the presence of the virus.

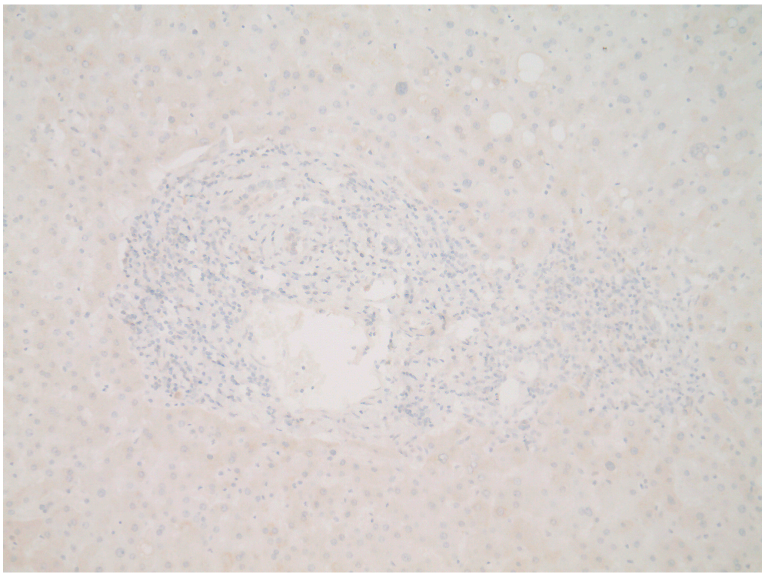

Figure S2. *Liver*: Chronic hepatitis, 100x magnification. Immunohistochemistry for SARS-CoV-2 nucleocapsid protein shows no staining, indicating the absence of viral infection in the hepatic tissue.
